# Considering Multiecosystem Trade‐Offs Is Critical When Leveraging Systematic Conservation Planning for Restoration

**DOI:** 10.1111/gcb.70020

**Published:** 2025-01-17

**Authors:** Nicholas J. Van Lanen, Courtney J. Duchardt, Liba Pejchar, Jessica E. Shyvers, Cameron L. Aldridge

**Affiliations:** ^1^ U.S. Geological Survey Fort Collins Science Center Fort Collins Colorado USA; ^2^ Oklahoma State University Stillwater Oklahoma USA; ^3^ Colorado State University Fort Collins Colorado USA; ^4^ The Nature Conservancy Global Protect Oceans, Lands and Water Fort Collins Colorado USA

**Keywords:** *Conservation Interactions Principle*, cross‐realm, ecotone, multi‐ecosystem management, optimization, prioritization, restoration, systematic conservation planning, trade‐offs

## Abstract

Conservationists are increasingly leveraging systematic conservation planning (SCP) to inform restoration actions that enhance biodiversity. However, restoration frequently drives ecological transformations at local scales, potentially resulting in trade‐offs among wildlife species and communities. The *Conservation Interactions Principle* (CIP), coined more than 15 years ago, cautions SCP practitioners regarding the importance of jointly and fully evaluating conservation outcomes across the landscape over long timeframes. However, SCP efforts that guide landscape restoration have inadequately addressed the CIP by failing to tabulate the full value of the current ecological state. The increased application of SCP to inform restoration, reliance on increasingly small areas to sustain at‐risk species and ecological communities, ineffective considerations for the changing climate, and increasing numbers of at‐risk species, are collectively intensifying the need to consider unintended consequences when prioritizing sites for restoration. Improper incorporation of the CIP in SCP may result in inefficient use of conservation resources through opportunity costs and/or conservation actions that counteract one another. We suggest SCP practitioners can avoid these consequences through a more detailed accounting of the current ecological benefits to better address the CIP when conducting restoration planning. Specifically, forming interdisciplinary teams with expertise in the current and desired ecosystem states at candidate conservation sites; improving data availability; modeling and computational advancements; and applying structured decision‐making approaches can all improve the integration of the CIP in SCP efforts. Improved trade‐off assessment, spanning multiple ecosystems or states, can facilitate efficient, proactive, and coordinated SCP applications across space and time. In doing so, SCP can effectively guide the siting of restoration actions capable of promoting the full suite of biodiversity in a region.

## Graphical abstract

Restoration frequently drives ecological transformations at local scales, potentially resulting in trade‐offs among wildlife species and communities. Improperly evaluating how the current system or state contributes to biodiversity conservation when spatially prioritizing restoration may result in inefficient use of resources through opportunity costs and/or conservation actions which counteract one another. We suggest systematic conservation planning practitioners can avoid these consequences through more detailed accounting of the current ecological benefits of an ecosystem or state when prioritizing restoration action.

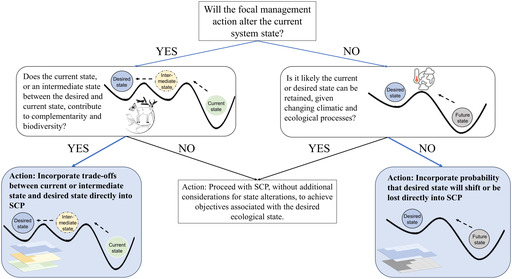

## 1 Introduction

Systematic conservation planning (SCP) is a widely applied and growing decision‐support approach within the field of conservation biology. Although early SCP efforts focused primarily on reserve design, SCP is increasingly implemented to guide restoration action. Recent examples of leveraging SCP to prioritize restoration action include, but are not limited to, effective sagebrush planting (*Artemisia* spp.; Duchardt et al. 2021); mechanically removing conifers (Van Lanen et al. 2023) to restore sagebrush communities; restoring seagrass, mangroves, and oyster reefs in estuarine systems (Gilby et al. 2021); enhancing or establishing new wetlands (Schleupner and Schneider 2013); and reforesting woodland communities (Justeau‐Allaire et al. 2020). These efforts have shown applying SCP to guide restoration efforts can enhance the likelihood of success, improve biological outcomes, enhance ecological resilience, and reduce restoration costs.

Although the application of SCP to prioritize restoration is relatively new and well‐intentioned, management action of any form is likely to result in biodiversity trade‐offs. The concept of trade‐offs, in an SCP context, was highlighted by Moilanen (2008) in his discussion of the “Conservation Interactions Principle” (CIP). The CIP states “conservation benefits of all conservation actions across the landscape should be evaluated jointly and account for long‐term consequences of interactions between actions.” Specifically, in defining the concept of “Multiple Alternative Conservation Actions,” Moilanen underscores that an action beneficial for one feature may be detrimental for another (i.e., “trade‐offs”).

Although ecological, social, and economic trade‐offs are frequently incorporated into SCP (Margules and Pressey 2000; Arponen et al. 2010; Kukkala and Moilanen 2013), incorporating ecological trade‐offs, associated with restoration efforts, across multiple ecosystems or ecological states (hereafter, “cross‐system”) is seldom done when prioritizing SCP restoration efforts. Instead, SCP efforts to guide restoration more frequently consider objectives associated with the desired ecological state and ignore the contribution of the existing ecological state to complementarity. This failure to consider cross‐system trade‐offs in SCP restoration efforts violates the established SCP principles, including CIP, and represents the motivation for this perspective piece. To address this shortcoming, we highlight how CIP may be violated when prioritizing restoration. We then provide some example systems and instances where SCP is liable to be incorrectly applied when prioritizing restoration. Finally, we share some suggestions and a decision tree to assist SCP practitioners in better addressing the CIP when prioritizing restoration action with SCP.

### 1.1 Balancing Costs and Benefits in SCP

Appropriately tabulating the benefits of conservation action, including those related to restoration, is done by estimating the change in conservation value with and without the action and then calculating the difference between these two scenarios (Maron, Rhodes, and Gibbons 2013). This accounting of benefits can then be extended to multiobjective optimization (Williams and Kendall 2017), a subfield of multicriteria decision‐making, which has been used to specifically address inherent trade‐offs in management. For instance, multiobjective optimization has been used to maximize game animal populations while minimizing crop damage (Williams and Kendall 2017) or to balance populations of multiple grassland species with disparate habitat requirements (van Teeffelen et al. 2008). Applications of SCP to guide conservation action have extended this idea of trade‐offs to incorporate the value of human relationships (Sowman and Sunde 2018), economic costs (Naidoo et al. 2006), threats to the system (Maron, Rhodes, and Gibbons 2013), risks associated with uncertainties and correlations of expected costs and benefits (Eaton et al. 2019), and ecosystem services (Mu et al. 2022). More recently, SCP efforts have even begun to consider the terrestrial‐aquatic interface (frequently referred to as “cross‐realm”), extending conservation objectives and utilities of terrestrial management and protection to incorporate effects on aquatic systems (including estuarine, freshwater, and marine) and vice versa (Hazlitt et al. 2010; Klein et al. 2012; Tulloch et al. 2021).

Despite these laudable efforts to incorporate multifaceted costs and benefits of conservation actions, we found few examples in the literature of SCP efforts that incorporate cross‐system trade‐offs associated with restoration within a strictly terrestrial or aquatic realm. In other words, when modification or restoration of a given ecosystem would lead to a new ecosystem state (a “zero‐sum game”), how do we decide if the current or proposed ecosystem state is more valuable when both could naturally occur in that region? For example, SCP efforts aimed at bolstering grassland bird populations may prioritize the management of shrub‐encroached sites, employing prescribed fire to reduce shrub cover. Such an effort may tabulate the expected increases in grassland‐associated wildlife populations, expected risk of accidental fire damage to structures in the area, and economic costs of safely introducing fires to various sites. However, only rarely have we found instances where SCP efforts to guide restoration considered the full ecological value of the “initial state”, in this case, the value of shrub‐encroached sites beyond their potential to be restored to grassland habitat. Shrub‐encroached sites may provide important shrubland habitat for wildlife but, if current ecological value is only viewed through the lens of value to grassland‐associated species, costs and benefits incorporated in the SCP effort may not represent the comprehensive cross‐system ecological outcomes. This is especially concerning if the “initial state” would have historically occurred within the region. Such oversights when conducting SCP for restoration are liable to violate the CIP and fall short of achieving complementarity, a stated goal of SCP.

In this example, prioritization efforts could effectively address the CIP, and potential detrimental effects of restoration action on another feature, if expected changes to shrubland‐associated wildlife species postmanagement are explicitly incorporated into the SCP effort. For instance, expected gains to grassland‐associated species could be maximized while transformations of important shrubland habitats could be minimized. In this instance, failing to consider the existing value of shrubland habitat to associated species may fail to account for the true biological costs and benefits of conservation action across space. However, more rigorous cross‐system application of the CIP, which fully incorporates the current biological value of the system, has received little attention from SCP practitioners to date, despite increasing efforts to do so using other conservation prioritization and management tools (Johnson et al. 2015; Romañach et al. 2022).

### 1.2 Why Consider Cross‐System Objectives When Prioritizing Restoration?

Considering the cross‐system effects of restoration action is of increasing importance for several reasons. First, escalating use of SCP software to inform restoration increases the likelihood that practitioners may violate the CIP by failing to account for undesired cross‐system trade‐offs. Second, as anthropogenic development continues, offsets and mitigation efforts (including restoration) are frequently used to reduce and/or counteract biodiversity loss and environmental degradation (Koh, Hahn, and Boonstra 2019), resulting in additional restoration efforts designed to alter system states. When restoration leads to ecological transformation, biodiversity contributions of an area are liable to shift from communities associated with the initial state to communities associated with the new or “restored” state. When negative effects on the existing community are not fully tabulated, as directed under the CIP, SCP efforts may fail to enhance regional biodiversity. Perceived gains from restoration may be partially or entirely offset by costs to the existing community (Box 1: One Ecosystem Per Site). With the increasing use of SCP to guide restoration which bolsters biodiversity and offsets or mitigates undesired anthropogenic effects, the potential for inadequate accounting of ecological costs and benefits leading to suboptimal decision‐making also increases.

BOX 1 **One Ecosystem Per Site.**

Land conversion and anthropogenic development represent two major drivers of global biodiversity loss (Tilman et al. 2017) and limit the area available to support intact ecosystems (Theobald et al. 2020). Unique ecosystems and ecotones, collectively supporting a broad array of biodiversity, individually require adequate area to support viable plant and animal communities. As each location on Earth can only support one system state at any given time, the spatial arrangement, extent, and successional stage for which we manage can have cascading effects on biodiversity.

Example: Temperate rainforests in the Pacific Northwest region of North America experienced widespread deforestation in the later portion of the 19th century. This deforestation, particularly of old‐growth trees, was primarily implicated in the decline of the northern spotted owl ( *Strix occidentalis caurina* ; Anthony et al. 2006). In response to northern spotted owl population declines the subspecies was listed as threatened under the U.S. Endangered Species Act (U.S. Fish and Wildlife Service 1990), resulting in large‐scale efforts to manage for closed‐canopy forests with high tree density, height, and age (a). However, such management has been shown to reduce occupancy rates of numerous songbird species which rely upon more heterogeneous forests (b), including several rare species (White et al. 2013). Optimizing management for northern spotted owls without consideration of other rare species' habitat requirements may result in suboptimal decisions when considering the need for both closed‐canopy and heterogeneous forests to support biodiversity. However, systematic conservation planning (SCP) is well‐suited to optimize these competing biodiversity objectives.

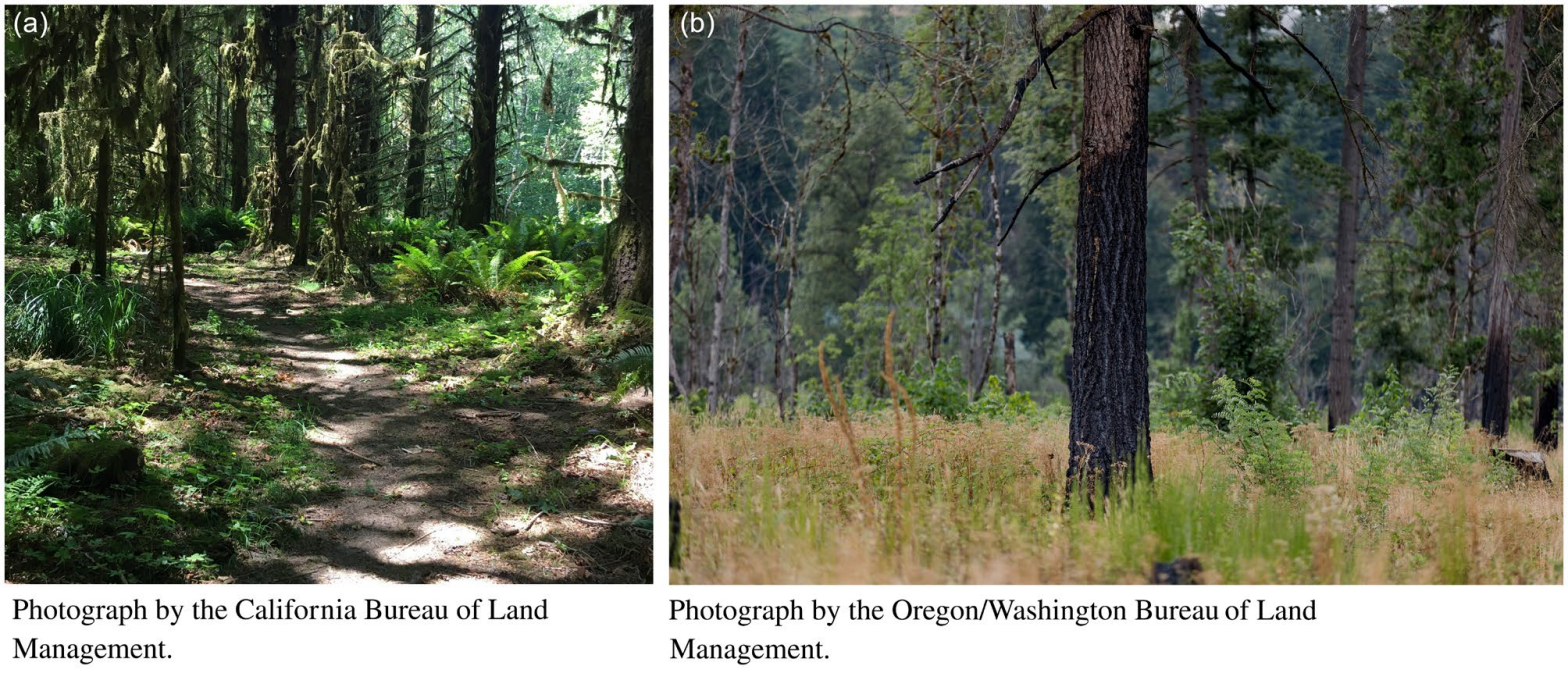

Changing climate is altering the distribution of species, communities, and ecosystems (Chen et al. 2011), further complicating the arithmetic of SCP. Climate change is accelerating ecological transitions in many regions (Boulanger et al. 2016; Osland et al. 2016), likely increasing overlap and/or encroachment of ecosystems. Given increasing frequency of ecological transitions, restoration planning may be improved by weighing the benefits of managing for historic ecosystems versus managing for ecosystems which are compatible with future climatic conditions (Grantham et al. 2011; Reside, Butt, and Adams 2017; Pressey et al. 2021; Vigo et al. 2024) (Box 2: Changing Climate and Spatial Transitions). Consideration of probabilities of natural and desired ecological transitions, timescales of these transitions, and the degree to which sites contribute to representation and complementarity of regional biodiversity, now and in the future, may represent important factors when weighting restoration actions in these situations. Furthermore, proactive and careful consideration to create habitat in advance of climatic shifts in species distributions may be critical to the effectiveness of SCP in these instances. Similarly, global expansion of the human footprint and development intensity equates to fewer unmodified areas supporting historical ecological function (Venter et al. 2016; Kennedy et al. 2019). If SCP practitioners desire the most comprehensive suite of biodiversity possible, across all ecoregions, SCP efforts may need to carefully consider the spatial arrangement and footprint of ecosystems. This will require difficult trade‐offs; any plot of land can only support a single ecosystem and successional stage at a particular point in time. As a result, place‐based decisions regarding what to manage for and where may become controversial when relevant parties' priorities differ (Box 1: One Ecosystem Per Site).

BOX 2 **Changing Climate and Spatial Transitions.**

Evidence suggests species' distributions are changing in response to a changing climate (Chen et al. 2011; Lenoir and Svenning 2014; Stephens et al. 2016). These shifting distributions can lead to difficult decisions regarding where and when to manage for a particular ecological state. Restoration ecology has a long history of relying upon historic conditions to inform management action (Society For Ecological Restoration International 2004). Continuing to base restoration on historic conditions may result in inefficient restoration efforts, with historically occurring plants exhibiting poor survival under new climatic conditions (Jellinek et al. 2020). Additionally, as ranges shift, at‐risk species may seek refugia within new climate envelopes which may be managed for historic conditions.

Example: The Cape Sable seaside sparrow ( *Ammospiza maritima mirabilis* ) is an endangered ground‐nesting bird inhabiting marl prairie, a freshwater‐inundated prairie ecosystem which dries each summer. Model predictions indicate rising water levels are liable to render current habitat for the Cape Sable seaside sparrow (a) unsuitable in the future (Romañach et al. 2022). Changes in management practices (e.g., hydrologic management of freshwater impoundments) may be employed to create alternate suitable habitat for the endangered sparrow; however, these management actions could have cascading negative effects for other priority species in the area. For instance, the endangered snail kite ( *Rostrhamus sociabilis plumbeus* ) requires shallow freshwater marshes, lakes, ponds, and impoundments where they forage for prey (b; Kitchens et al. 2002). Draining these freshwater wetlands could create suitable habitat for the sparrow but would directly reduce kite habitat. Explicit consideration of which species to manage for and where may lead to more wholistic systematic conservation planning (SCP). Such an effort could help retain representation of all at‐risk biodiversity within the Everglades, despite a changing climate driving spatial transitions of saltwater, freshwater, and marl prairie ecosystems.

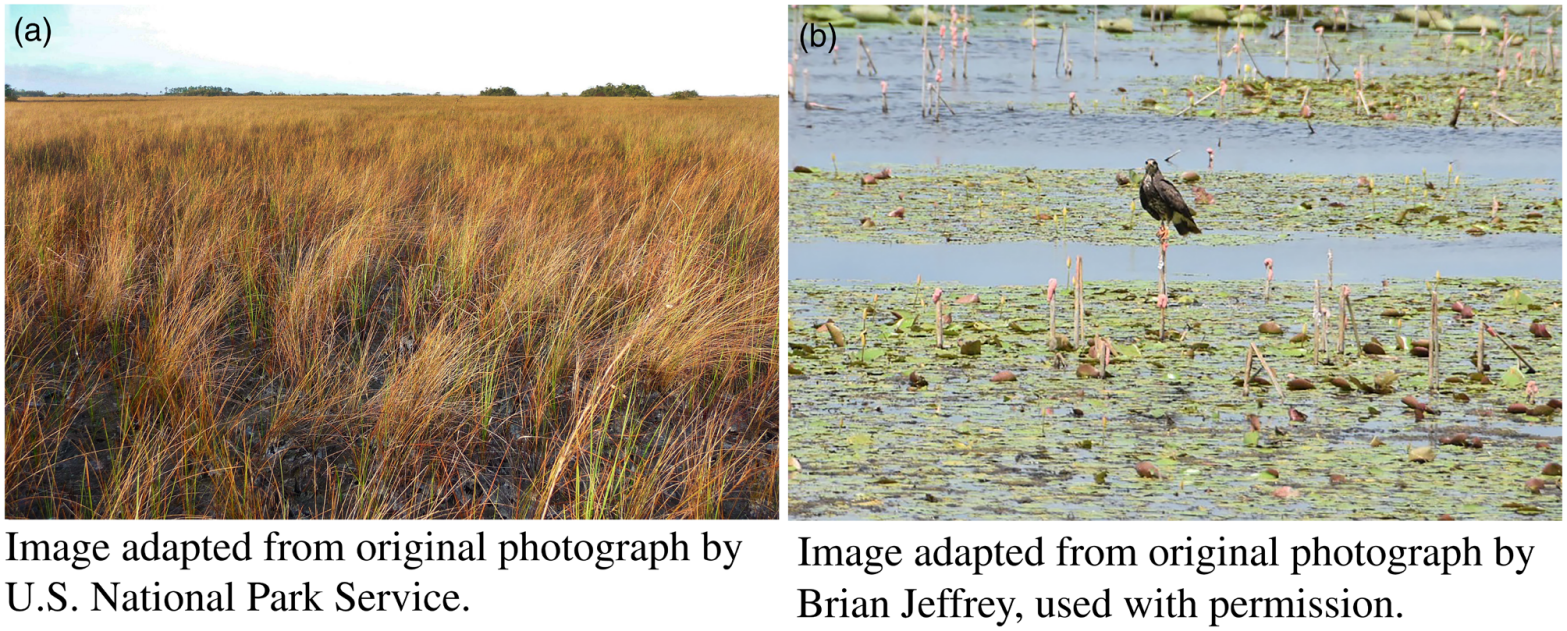

Threats to biodiversity are prevalent across the globe (Tilman et al. 2017), accelerating rates of extinction of vertebrate species (Ceballos et al. 2015). Thus, the possibility of undesired trade‐offs from restoration action negatively affecting an at‐risk species or system is likely to increase over time. To better address this challenge, SCP efforts can be improved through the accurate tabulation of costs and benefits, accounting not only for the benefits of management to target species but also for undesired consequences to other taxa and systems, particularly those existing within potential restoration sites (Box 3: Which Species to Prioritize?).

BOX 3 **Which Species to Prioritize?**

As the number of at‐risk species increases globally so does the likelihood that species with contrasting habitat requirements will occur in areas of sympatry. Land management decisions can greatly influence habitat suitability, and decisions regarding which species to prioritize are liable to influence population viability and biodiversity. Directly weighing the costs and benefits of ecological transformations may result in land management efforts that benefit the full suite of priority species.

Example: Sagebrush ecosystems (*Artemisia spp*.) in the western United States have become increasingly converted, disturbed, and fragmented, ultimately resulting in the decline of wildlife species associated with them. In particular, the greater sage‐grouse ( *Centrocercus urophasianus* ; hereafter, “sage‐grouse”) has experienced long‐term population declines to the point where the species was proposed for listing under the U.S. Endangered Species Act (US Fish and Wildlife Service 2013). Efforts to recover sage‐grouse include the removal of coniferous trees (a) from areas supporting sagebrush understories. In the central and southern portions of the sage‐grouse range, trees associated with pinyon‐juniper woodlands (including juniper [*Juniperus spp*.] and pinyon pine [ *Pinus edulis* and *Pinus monophylla* ]) are frequently targeted for these conifer removal efforts (Reinhardt et al. 2020). Conifer removal to restore sagebrush ecosystems is primarily occurring at pinyon‐juniper and sagebrush ecotones (b), which may also represent important habitat for the pinyon jay ( *Gymnorhinus cyanocephalus* ; Boone et al. 2021; Van Lanen et al. 2023), another species recently proposed for listing under the Endangered Species Act (Defenders of Wildlife, 2022). Therefore, systematic conservation planning (SCP) efforts to optimize conifer removal, which fail to consider negative effects to the pinyon jay may result in undesired outcomes for the pinyon jay (Van Lanen, Monroe, and Aldridge 2023), ultimately resulting in biodiversity loss.

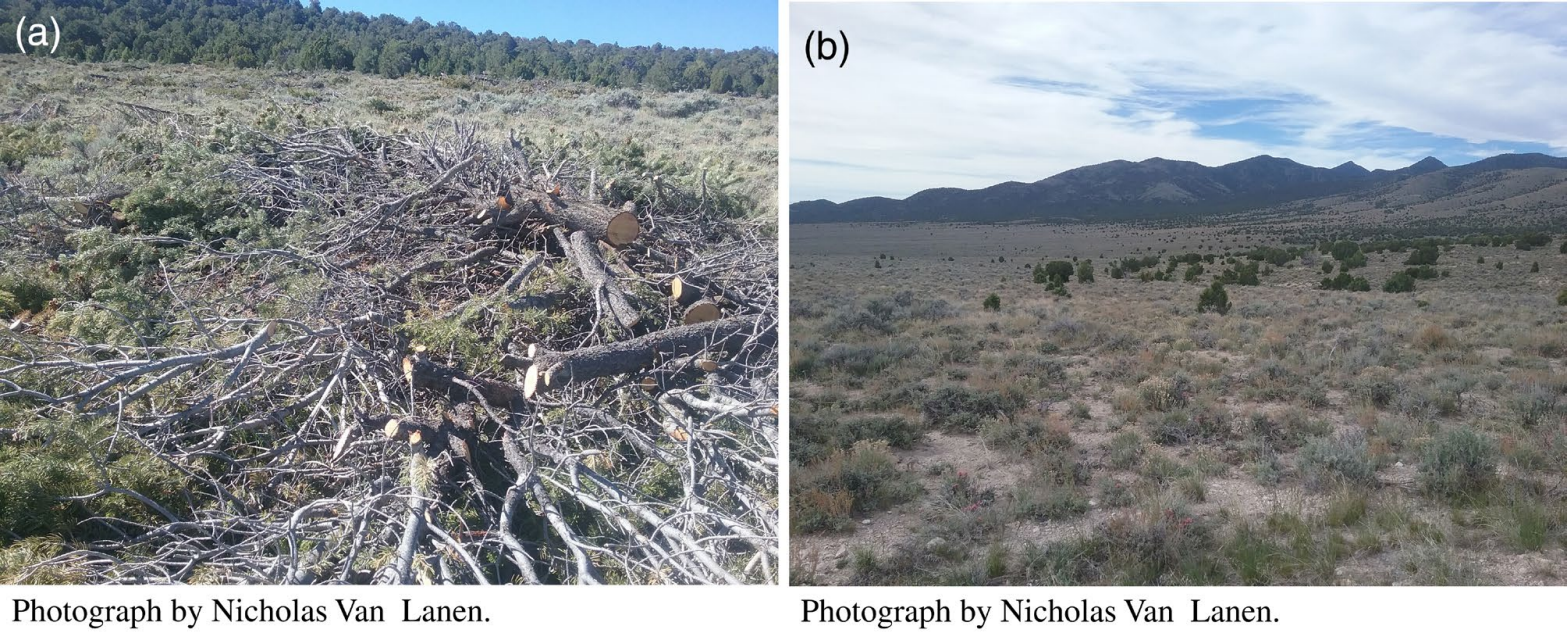

### 1.3 Consequences of Incomplete SCP Trade‐Off Accounting

When restoration prioritization improperly accounts for undesired effects of conservation action, restoration may insufficiently address complementarity and result in biased SCP solutions (Van Lanen et al. 2023). Furthermore, when undesired effects are not incorporated, restoration of one area may offset actions elsewhere when objectives differ (Boxes 1–, 3). Yet, despite the recent wave of SCP applications for guiding ecological transformations in the literature (Ribeiro and Atadeu 2019; Villarreal‐Rosas et al. 2020; Gebre et al. 2021), there remains a paucity of studies that directly evaluate these potential undesired trade‐offs.

### 1.4 The Challenges of Incorporating Multiple Ecosystems in SCP

Prioritization efforts spanning multiple jurisdictions and ecosystems will inherently require additional SCP inputs and thereby increase the complexity of future SCP efforts. Additional inputs will also necessitate more information exchange and coordination across roles, agencies, and countries. Furthermore, increasing the complexity of restoration planning through additional optimization inputs is likely to result in cascading uncertainty, reduced precision of SCP solutions, and more variability regarding predicted outcomes due to imperfect knowledge of species responses to restoration action. As more trade‐offs are considered, optimal portfolios developed through SCP may result in intermediate outcomes, where all objectives are marginally met but none are achieved as efficiently and effectively as if they were the sole objective. In some instances, such as when trying to save a critically endangered species, prioritization which incorporates many objectives but only marginally addresses any of them may be undesirable. Finally, the increased optimization complexity and larger spatial extents that are required to facilitate more wholistic planning efforts will increase computational hardware and software requirements, potentially making SCP less tractable for many conservation planners. We note similar challenges have been described in other recent efforts (Law et al. 2021; Iglesias et al. 2024); however, we wish to reiterate them here, with an emphasis on how these challenges are liable to manifest when leveraging SCP to guide restoration.

### 1.5 Recommendations for Addressing Multiecosystem SCP Challenges

**FIGURE 1:**
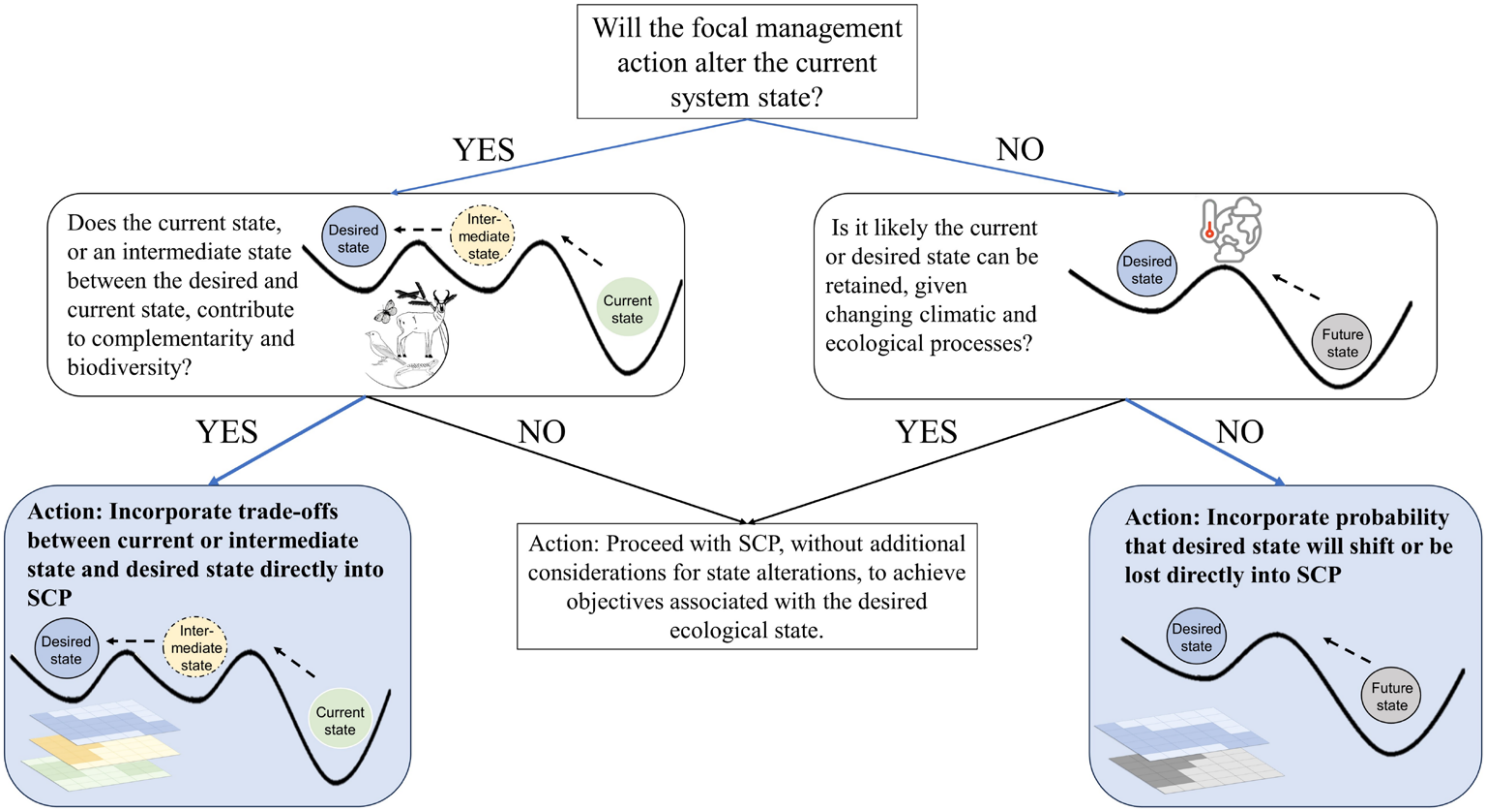
A suggested decision tree to help systematic conservation planning (SCP) practitioners consider the need for more detailed integration of the Conservation Interactions Principle (CIP) when prioritizing restoration action. Blue arrows point to questions and actions where CIP can be integrated. Boxes with bold text highlight the inclusion of CIP in SCP.

Despite the above challenges, the SCP toolbox is already well‐equipped to address cross‐system trade‐offs when prioritizing restoration. In Figure 1, we provide a suggested decision tree to help practitioners assess the need for formal CIP integration into SCP for restoration. Specifically, we urge practitioners to consider whether the SCP effort is prioritizing ecological transformation (as opposed to ecological protection), the probability of successfully achieving the desired transformation, and the degree to which current or intermediate ecological states contribute to the comprehensiveness and representation of regional biodiversity. It is this additional step of considering current or intermediate states' contribution to regional biodiversity that has been insufficiently addressed in recent work. We suggest the inclusion of this step can help practitioners view SCP through a broader and more wholistic lens, leading to potentially improved restoration outcomes that align with the CIP. We are encouraged that existing SCP frameworks provide ample opportunities to explicitly evaluate cross‐system trade‐offs to current or intermediate ecological states by directly incorporating additional SCP inputs (Watts et al. 2009; Hanson et al. 2021). We recognize effectively incorporating trade‐offs across ecosystems will require additional collaboration, compromise, and information sharing.

Fortunately, interdisciplinary science is on the rise and scientists are becoming accustomed to working as part of larger and more diverse teams (Haeussler and Sauermann 2020). We also note scientific journals are encouraging improved data availability (McNutt 2016), which may help alleviate some limitations regarding the sharing of data across agencies (e.g., when data are proprietary or when data sharing agreements can pose barriers). Furthermore, developments in species distribution modeling approaches and software (Guillera‐Arroita 2017; Kellner et al. 2023) have made feature layer production easier for researchers and conservation planners. Similarly, advancements in high performance computing, high‐throughput computing, cloud computing (including the Marxan planning platform [MaPP], https://marxansolutions.org/marxanmapp/), and commercial solvers make complex and memory intensive optimization procedures, spanning multiple ecosystems, easier to implement. Finally, the use of structured decision‐making frameworks can help resolve relevant party disagreements and address competing interests in an equitable way (Gregory et al. 2012). For instance, the PrOACT process (Hammond et al. 1999) emphasizes identification of the conservation problem, project objectives, alternative actions, and expected consequences. Such an approach can transparently address restoration objectives of multiple parties, lead to a more inclusive effort, and ultimately increase buy‐in regarding SCP products (Sink et al. 2023).

## 2 Conclusion

We see the widespread omission of cross‐system opportunity costs and direct trade‐offs to be an unfortunate oversight in some ongoing SCP practices and a concerning limitation of ecological restoration more broadly. We suggest a more accurate and representative accounting of costs and benefits to address CIP (Moilanen 2008; Maron, Rhodes, and Gibbons 2013) can benefit efforts to conserve and promote a more complete suite of biodiversity across ecosystems. The decision tree we provide in Figure 1 can serve as a tool for practitioners to help work through the potential need to more closely integrate the CIP into SCP of restoration efforts. We recognize there are numerous challenges including added complexity, disparate data sources, and multiple relevant parties' objectives when optimizing restoration action across ecosystems. However, we are pleased to highlight the fields of SCP and decision science are already largely equipped to overcome these challenges in a transparent and equitable way. We are also encouraged to see a recent example of SCP which addresses cross‐system objectives when prioritizing restoration (Iglesias et al. 2024). We suggest this paper may serve as an additional resource for SCP practitioners wishing to enhance the CIP when prioritizing restoration.

In this perspective piece, we focused on balancing biological outcomes of restoration across multiple ecosystems; however, we do not advocate for the removal of other, valuable additions to conservation planning which have been put forward. Some recent additions into SCP procedures include incorporating human relationships, monetary costs, political interests, uncertainty, and ecosystem services into prioritization efforts. We also agree with prior suggestions to track effectiveness and outcomes to facilitate learning in the form of “adaptive conservation planning” (see Bottrill and Pressey 2012), and support the inclusion of the five types of capital (natural, financial, social, human, and institutional; Bottrill and Pressey 2012). However, we urge for the extension of these elements to all ecosystems and states affected by restoration actions. Studies have shown that prioritization outcomes are strongly influenced by the optimization inputs (i.e., costs and conservation features) and can yield widely divergent optimized solutions (Evans et al. 2015; Duchardt et al. 2021; Van Lanen et al. 2023; Wang et al. 2023). Therefore, we suggest SCP practitioners may wish to expand trade‐off assessment to consider multiple ecosystems and states. Doing so will better address the CIP, thereby facilitating efficient, proactive, and coordinated SCP applications to support restoration outcomes that promote the full suite of biodiversity in a region.

## Author Contributions

**Nicholas J. Van Lanen:** conceptualization, investigation, methodology, writing – original draft, writing – review and editing. **Courtney J. Duchardt:** conceptualization, writing – review and editing. **Liba Pejchar:** conceptualization, writing – review and editing. **Jessica E. Shyvers:** conceptualization, writing – review and editing. **Cameron L. Aldridge:** conceptualization, project administration, supervision, writing – review and editing.

## Conflicts of Interest

The authors declare no conflicts of interest.

## Data Availability

No data were used in the development of this manuscript.
